# Comparison of the Walking Hip Spica Cast and the Conventional Spica Cast in Femoral Shaft Fractures in Children Under Five Years of Age

**DOI:** 10.7759/cureus.93027

**Published:** 2025-09-23

**Authors:** Abdur Rauf, Khalid Khan, Muhammad Tanveer, Zawar Ahmad, Muhammad Tayyab, Asif Afridi, Muhammad Javed Khan

**Affiliations:** 1 Orthopaedic Surgery, Buner Medical Complex Daggar, Buner, PAK; 2 Orthopaedics, Medical Teaching Institution (MTI) Mardan Medical Complex, Bacha Khan Medical College, Mardan, PAK; 3 Trauma and Orthopaedics, Royal Stoke University Hospital, University Hospitals of North Midlands, Stoke-on-Trent, GBR; 4 Trauma and Orthopaedics, Kettering General Hospital NHS Foundation Trust, Kettering, GBR; 5 Trauma and Orthopaedics, Bradford Teaching Hospitals NHS Foundation Trust, Bradford, GBR; 6 Trauma and Orthopaedics, Hayatabad Medical Complex Peshawar, Peshawar, PAK; 7 Trauma and Orthopaedics, University Hospitals Birmingham NHS Foundation Trust, Birmingham, GBR; 8 Paediatric Surgery, Bacha Khan Medical College, Mardan, PAK

**Keywords:** conventional hip spica, femoral shaft fracture, fracture healing, parental satisfaction, pediatric orthopedics, walking spica cast

## Abstract

Background

Femoral shaft fractures are common in children under five, and conservative management with hip spica casting remains the standard of care.

Objective

The main objective of this study is to compare clinical, radiological, functional, and caregiver-related outcomes between walking hip spica and conventional hip spica casts in children under five years of age with femoral shaft fractures.

Methodology

This prospective observational study was conducted at the Department of Orthopaedic Surgery, Mardan Medical Complex (MMC), Mardan, Pakistan, from January 2023 to December 2024. A total of 94 children aged 1-5 years with closed, isolated femoral shaft fractures were enrolled. Patients were divided into two groups: 47 children (50%) received a walking hip spica cast (Group A), and 47 children (50%) received a conventional hip spica cast (Group B). Follow-up evaluations were performed at 2, 4, 6, and 12 weeks to assess radiological union, complications, functional milestones, and parental satisfaction. Data were analyzed using IBM SPSS Statistics for Windows, Version 25 (Released 2017; IBM Corp., Armonk, NY, USA), with significance set at p < 0.05.

Results

Radiological union at 12 weeks was achieved in all 47 patients (100%) in Group A, and 46 patients (97.87%) in Group B. Mean time to union was significantly shorter in Group A (6.28 ± 1.04 weeks) versus Group B (6.74 ± 1.21 weeks, p = 0.021). Complications occurred in five patients (10.64%) in Group A and 12 patients (25.53%) in Group B (p = 0.048). Functional recovery at six weeks (walking unassisted) was observed in 36 patients (76.60%) in Group A and 30 patients (63.83%) in Group B. High parental satisfaction was reported by 42 parents (89.36%) in Group A versus 28 parents (59.57%) in Group B (p = 0.001).

Conclusion

Walking hip spica casts demonstrated better clinical, functional, and caregiver satisfaction outcomes than conventional spica casts in young children with femoral shaft fractures.

## Introduction

One of the most frequent long bone injuries in children, especially in those under five, is a femoral shaft fracture [1,2]. Because the bones in this age range are generally soft and malleable, low-energy trauma, such as falls from heights or domestic mishaps, is the main cause of these fractures [3]. Due, in great part, to their exceptional bone remodeling capacity and the avoidance of surgical risks, non-operative therapy is still the accepted standard of care for the majority of femoral shaft fractures in children under five [4]. Because it provides enough immobilization to promote bone repair, the traditional hip spica cast has long been the mainstay of conservative therapy [5].

The traditional hip spica cast has a number of disadvantages despite its efficacy [6]. The kid becomes totally immobile from the chest to the toes, which may cause problems with daily care and hygiene, skin issues, muscular atrophy, and caregiver stress [7]. Furthermore, a child's mental development, at a crucial period of growth and independence, may be adversely affected by extended immobility [8].

The walking hip spica cast is a modification that has gained popularity recently, with the goal of enhancing comfort and functioning without sacrificing the healing process [9]. Partial mobility is made possible by this cast, especially ambulation with assistance, which may lead to a quicker functional recovery and an enhanced quality of life [10]. While preserving the mechanical stability necessary for fracture healing, the walking hip spica may lessen the problems related to total immobility by permitting weight-bearing and restricted motion [11]. Although walking spica casts are becoming more and more popular, there is a dearth of high-quality comparative data evaluating their effectiveness in comparison to traditional ones, particularly with regard to fracture union, complication rates, parental satisfaction, and functional outcomes in children under five [12].

It is crucial to assess if new developments, like the walking spica cast, provide quantifiable therapeutic advantages over the conventional approach when treatment approaches change. By examining and contrasting the results of these two casting methods in young children with femoral shaft fractures, this research aims to close this gap.

Research objective

The main objective of this study is to compare the clinical and functional outcomes of walking hip spica cast versus conventional spica cast, in the management of femoral shaft fractures in children under five years of age.

## Materials and methods

Study design and setting

This prospective observational study was conducted at the Department of Orthopaedic Surgery, Mardan Medical Complex (MMC) in Mardan, Pakistan, from January 2023 to December 2024.

Inclusion and exclusion criteria

The research included children with isolated, closed femoral shaft fractures who were between the ages of one and five. Eligible patients had to show up within 72 hours of the accident and receive non-operative treatment with any kind of hip spica cast. Before being included, parents or legal guardians gave their informed permission. Patients with pathological fractures, open fractures, concomitant polytrauma, metabolic bone illnesses, neuromuscular disorders that impair movement, or those who were lost to follow-up were not included in the study. All patients were followed throughout the study period, and no post-enrollment attrition occurred.

Sample size

Convenience sampling was used to choose 94 patients from the eligible pediatric orthopedic outpatient and inpatient population. Because of the single-center design and the intention to enroll all consecutive patients who met the inclusion criteria during the course of the two-year study period, convenience sampling was used. Since the research was exploratory in nature and sought to replicate actual clinical procedures in the conservative care of femoral shaft fractures in children under five, no formal a priori power or sample size calculation was carried out.

A post-hoc power analysis was performed to assess the adequacy of the achieved sample size. Assuming a medium effect size (Cohen’s d = 0.5) and a significance level of 0.05, the final sample of 94 patients (47 per group) provided a statistical power of approximately 80%, which is generally considered acceptable for detecting clinically meaningful differences.

Furthermore, compared to previous observational studies conducted in comparable pediatric orthopedic settings, the final sample size is comparable or larger [13,14]. The Discussion section has recognized this methodological restriction. Based on the type of cast used, the recruited patients were split into two groups: 47 patients received a walking hip spica cast (Group A) and 47 patients received a traditional hip spica cast (Group B).

Data collection

A standardized proforma, created by the lead investigator in collaboration with senior orthopedic surgeons, was used to gather data (Table 7, see Appendix). The proforma documented patient age, gender, fracture pattern, mechanism, and side of injury; type of spica cast (traditional or walking); and duration of immobilization. At 2, 4, 6, and 12 weeks after the installation of the cast, follow-up evaluations were carried out. Time to clinical and radiological fracture union, cast-related complications (such as skin excoriation, pressure sores, cast breakage, re-displacement, or malunion), and functional recovery indicators (such as the child's capacity to sit independently, stand with assistance, and walk following cast removal) were considered follow-up outcomes. To minimize observer bias, radiological union and functional recovery were independently assessed by two orthopedic consultants not involved in the index procedure, with disagreements resolved by consensus. In addition, parental satisfaction and ease of care were documented to assess the practicality of each casting technique.

Overall outcomes were categorized into three groups (excellent, satisfactory, and poor) based on a composite evaluation of fracture healing, functional recovery, and complication profile.

Excellent Outcome

Complete fracture union within 8-10 weeks, absence of complications (such as re-displacement, malunion, or significant skin/cast problems), and achievement of key functional milestones (independent sitting by two weeks, standing with support by four weeks, and independent walking by 12 weeks).

Satisfactory Outcome

Fracture union achieved but delayed beyond 10 weeks, or presence of minor complications (skin excoriation and mild cast breakage) without functional impairment; functional milestones achieved, but with some delay compared to the expected timeframe.

Poor Outcome

Delayed or non-union requiring extended immobilization (>12 weeks) or re-casting; occurrence of major complications (significant malunion, persistent re-displacement, or pressure sores); and failure to achieve independent walking by 12 weeks.

Statistical analysis

IBM SPSS Statistics for Windows, Version 25 (Released 2017; IBM Corp., Armonk, NY, USA) was used for data entry and analysis. For the demographic and clinical data, descriptive statistics such as means, standard deviations, frequencies, and percentages were computed. The independent samples t-test for continuous variables and the Chi-square test for categorical variables were used to compare the two groups. p-values below 0.05 were regarded as statistically significant.

Ethical approval

Ethical approval for the research was obtained from the Institutional Review Board (IRB) of Bacha Khan Medical College (BKMC), which is affiliated with Mardan Medical Complex (MMC), Mardan. The ethical number is 721/DOS/BKMC, dated December 13, 2022. Written informed consent was obtained from the parents or guardians of all participants. Anonymity was strictly maintained throughout the study, and all data were fully anonymized to ensure confidentiality.

## Results

The mean age of the 94 patients (47 in each group) was 3.08 ± 1.24 years for the conventional group and 3.21 ± 1.12 years for the walking spica group (Table 1). While 30 patients (63.83%) were male and 17 patients (36.17%) were female in the conventional group, 28 patients (59.57%) were male and 19 patients (40.43%) were female in the walking spica group. The right side was injured in 26 patients (55.32%) in the conventional group and 24 patients (51.06%) in the walking group. Transverse fractures occurred in 22 patients (46.81%) in the walking spica group and 24 patients (51.06%) in the conventional group; oblique/spiral fractures occurred in 25 patients (53.19%) and 23 patients (48.94%), respectively. A fall from a height was the most frequent cause of injury, occurring in 35 patients (74.47%) in the walking group and 36 patients (76.60%) in the conventional group. The walking group experienced a significantly shorter mean duration of immobilization (39.62 ± 4.85 days) than the conventional group (44.15 ± 5.11 days, p = 0.017).

**Table 1:** Demographic and Clinical Characteristics of Patients *p < 0.05 is considered statistically significant. Values are presented as mean ± standard deviation (SD) or n (%). An independent samples t-test was used for continuous variables; the Chi-square test was used for categorical variables.

Variable	Subcategory	Walking Spica (n; %)	Conventional Spica (n; %)	p-value (Test)
Age (years)	Mean ± SD	3.21 ± 1.12	3.08 ± 1.24	t = 0.62, p = 0.538 (Independent t-test)
Gender	Male	28 (59.57)	30 (63.83)	χ² = 0.166, p = 0.684
	Female	19 (40.43)	17 (36.17)	
Side of Injury	Right	26 (55.32)	24 (51.06)	χ² = 0.259, p = 0.611
	Left	21 (44.68)	23 (48.94)	
Fracture Pattern	Transverse	22 (46.81)	24 (51.06)	χ² = 0.501, p = 0.479
	Oblique/Spiral	25 (53.19)	23 (48.94)	
Mechanism of Injury	Fall from height	35 (74.47)	36 (76.60)	χ² = 0.084, p = 0.772
	Household accident	12 (25.53)	11 (23.40)	
Duration of Immobilization (days)	Mean ± SD	39.62 ± 4.85	44.15 ± 5.11	t = -2.45, p = 0.017*

Fracture healing outcomes favored the walking spica group, as presented in Table 2. The mean time to radiological union was significantly shorter in the walking spica group (6.28 ± 1.04 weeks), compared to the conventional group (6.74 ± 1.21 weeks, p = 0.021). At six weeks, 39 patients (82.98%) in the walking group achieved radiological union, versus 34 patients (72.34%) in the conventional group. By 12 weeks, union was achieved in all 47 patients (100%) in the walking group and 46 patients (97.87%) in the conventional group. Re-displacement occurred in two patients (4.26%) in the walking group, compared to five patients (10.64%) in the conventional group, while malunion was observed in one patient (2.13%) versus three patients (6.38%), respectively. Although not statistically significant, the trend consistently favored better healing outcomes with the walking spica cast.

**Table 2:** Fracture Healing Outcomes *p < 0.05 is considered statistically significant. Values are presented as mean ± SD or n (%). An independent samples t-test was used for mean time to union, and the Chi-square test was used for categorical outcomes.

Outcome	Walking Spica (n; %)	Conventional Spica (n; %)	p-value (Test)
Mean Time to Union (weeks ± SD)	6.28 ± 1.04	6.74 ± 1.21	t = -2.35, p = 0.021*
Radiological Union at 6 Weeks	39 (82.98)	34 (72.34)	χ² = 1.50, p = 0.221
Radiological Union at 12 Weeks	47 (100.00)	46 (97.87)	χ² = 1.02, p = 0.313
Re-displacement	2 (4.26)	5 (10.64)	χ² = 1.41, p = 0.236
Malunion	1 (2.13)	3 (6.38)	χ² = 1.04, p = 0.307

Cast-related complications were significantly fewer in the walking spica group, as detailed in Table 3. Among the 47 patients in each group, only five patients (10.64%) in the walking spica group experienced any complication, compared to 12 patients (25.53%) in the conventional group (p = 0.048). Specifically, skin excoriation occurred in three patients (6.38%) in the walking group, versus eight patients (17.02%) in the conventional group; pressure sores were noted in two patients (4.26%) vs. six patients (12.77%); and cast breakage was reported in one patient (2.13%) compared to four patients (8.51%). Although individual complication rates did not reach statistical significance, the overall complication burden was notably lower in the walking spica group, indicating better tolerability and cast integrity.

**Table 3:** Cast-Related Complications *p < 0.05 is considered statistically significant. Data are presented as n (%). Comparisons were made using the Chi-square test.

Complication Type	Walking Spica (n; %)	Conventional Spica (n; %)	p-value (Test)
Skin Excoriation	3 (6.38)	8 (17.02)	χ² = 2.58, p = 0.109
Pressure Sores	2 (4.26)	6 (12.77)	χ² = 2.16, p = 0.140
Cast Breakage	1 (2.13)	4 (8.51)	χ² = 1.91, p = 0.167
Any Complication	5 (10.64)	12 (25.53)	χ² = 3.90, p = 0.048*

Functional recovery was significantly faster in the walking spica group, as illustrated in Table 4. By the second week, 45 patients (95.74%) in the walking group were able to sit independently, compared to 38 patients (80.85%) in the conventional group (p = 0.030). At four weeks, 40 patients (85.11%) in the walking group could stand with support, versus 29 patients (61.70%) in the conventional group (p = 0.010). By six weeks, 36 patients (76.60%) in the walking group were walking unassisted, compared to 30 patients (63.83%) in the conventional group (p = 0.031). At 12 weeks, nearly all patients in both groups were walking independently, with 46 patients (97.87%) in the walking group and 42 patients (89.36%) in the conventional group (p = 0.093). These results highlight the accelerated functional milestones achieved with the walking hip spica cast.

**Table 4:** Functional Recovery Indicators Post-cast Removal *p < 0.05 is considered statistically significant. Data are presented as n (%). Functional outcomes were compared using the Chi-square test.

Functional Outcome	Walking Spica (n; %)	Conventional Spica (n; %)	p-value (Test)
Sitting Independently (2 weeks)	45 (95.74)	38 (80.85)	χ² = 4.70, p = 0.030*
Standing With Support (4 weeks)	40 (85.11)	29 (61.70)	χ² = 6.61, p = 0.010*
Walking Unassisted (6 weeks)	36 (76.60)	30 (63.83)	χ² = 4.64, p = 0.031*
Walking Independently (12 weeks)	46 (97.87)	42 (89.36)	χ² = 2.83, p = 0.093

Parental satisfaction was significantly higher in the walking spica group, as detailed in Table 5. A total of 42 parents (89.36%) in the walking spica group reported high levels of satisfaction, compared to only 28 parents (59.57%) in the conventional group (p = 0.001). Caregivers also found hygiene care more manageable with the walking spica, with just seven parents (14.89%) reporting difficulty, in contrast to 23 parents (48.94%) in the conventional group (p < 0.001). Furthermore, 44 parents (93.62%) in the walking group expressed willingness to choose the same cast again for future treatment, significantly higher than the 29 parents (61.70%) in the conventional group (p < 0.001). These findings underscore the practical and caregiving advantages of the walking spica cast in pediatric femoral shaft fracture management.

**Table 5:** Parental Satisfaction and Ease of Care *p < 0.05 is considered statistically significant. Data are presented as n (%). Comparisons were made using the Chi-square test.

Parameter	Walking Spica (n; %)	Conventional Spica (n; %)	p-value (Test)
High Parental Satisfaction	42 (89.36)	28 (59.57)	χ² = 10.65, p = 0.001*
Difficulty in Hygiene Care	7 (14.89)	23 (48.94)	χ² = 12.56, p < 0.001*
Preferred Same Cast Again (Yes)	44 (93.62)	29 (61.70)	χ² = 13.29, p < 0.001*

Outcomes were notably superior in the walking spica group, as shown in Table 6. Of the 47 patients in each group, 38 patients (80.85%) in the walking spica group achieved excellent outcomes, significantly higher than the 25 patients (53.19%) in the conventional group (p = 0.006). Satisfactory outcomes were observed in seven patients (14.89%) in the walking group and 15 patients (31.91%) in the conventional group. Poor outcomes were reported in only two patients (4.26%) treated with the walking spica, compared to seven patients (14.89%) in the conventional spica group. These findings suggest a clear advantage of the walking hip spica in improving overall clinical outcomes in young children with femoral shaft fractures.

**Table 6:** Overall Outcome Summary *p < 0.05 is considered statistically significant. Data are presented as n (%). Composite outcomes were classified as Excellent, Satisfactory, or Poor, based on predefined criteria, including time to union, complication profile, and functional recovery milestones. Composite outcomes were compared using the Chi-square test.

Composite Outcome	Walking Spica (n; %)	Conventional Spica (n; %)	p-value (Test)
Excellent Outcome	38 (80.85)	25 (53.19)	χ² = 7.63, p = 0.006*
Satisfactory Outcome	7 (14.89)	15 (31.91)	
Poor Outcome	2 (4.26)	7 (14.89)	

## Discussion

The goal of the current research was to evaluate the clinical and functional results of walking hip spica casts vs. traditional hip spica casts in the conservative treatment of femoral shaft fractures in children younger than five. According to our research, the walking spica cast offers significant benefits over the traditional spica in terms of healing duration, complication rates, functional recovery, and parental satisfaction.

In comparison to the conventional group (6.74 ± 1.21 weeks; p = 0.021), the walking spica group had a considerably shorter mean time to radiological union (6.28 ± 1.04 weeks). This is in line with other research showing that removable orthotic devices and functional casts promote efficient healing in young patients and enable early mobility and activity restoration [15]. Similar to our results, prior research found that the walking spica group's recovery period was somewhat shorter (5.8 weeks) than that of the traditional group (6.3 weeks), although the difference was not statistically significant [16].

The walking spica group saw a decreased incidence of malunion (2.13% walking vs. 6.38% conventional) and re-displacement (4.26% walking vs. 10.64% conventional), albeit these differences were not statistically significant. These results are corroborated by earlier research demonstrating that modified spica procedures, which provide partial movement without sacrificing fracture integrity, resulted in equivalent or fewer problems [11]. Importantly, at 12 weeks, union was attained in all walking spica patients (100%) and in 97.87% of the traditional group, indicating that both procedures were successful but that walking spica was superior in terms of speed and fewer complications. The walking group had considerably fewer cast-related difficulties (10.64%) than the traditional group (25.53%; p = 0.048). Among these were decreased incidences of cast breakage (2.13% vs. 8.51%), pressure sores (4.26% vs. 12.77%), and skin excoriation (6.38% vs. 17.02%). These results are consistent with earlier research findings that modified spica casts intended for limited mobility have better tolerance and fewer dermatological problems [17].

The walking spica group saw a much greater functional recovery. By the second week, 95.74% were able to sit on their own (compared to 80.85% in the conventional group); by the fourth week, 85.11% were able to stand with assistance (compared to 61.70%); and by the sixth week, 76.60% were able to walk without assistance (compared to 63.83%). These quicker milestones are consistent with findings from earlier research that showed early weight-bearing to be a driving force behind younger fracture patients’ faster motor development [18].

Along with fewer cleanliness issues (14.89% vs. 48.94%) and a higher readiness to reuse the same cast (93.62% vs. 61.70%), parental satisfaction was also considerably better in the walking spica group (89.36% vs. 59.57%; p = 0.001). As previously highlighted by Flynn et al. (2011), these results support the broader advantages of functional casting in lowering caregiver strain and enhancing quality of life during rehabilitation [11].

Strengths and limitations

This study’s prospective design, with systematic follow-up, is a key strength, ensuring consistent data collection and minimizing recall bias. By directly comparing two commonly used casting techniques in a pediatric orthopedic context, the research provides meaningful insights into functional recovery, caregiver satisfaction, and complication rates. The inclusion of standardized functional milestones and parent-reported outcomes further enriches the clinical evaluation. Independent assessment of radiographic and functional outcomes helped reduce observer bias, and complete follow-up with no post-enrollment attrition adds reliability to the findings.

Certain limitations must be acknowledged. The use of convenience sampling and the single-center design may restrict the generalizability of the results. Although the achieved sample size (n = 94) was comparable to or larger than prior observational studies, and a post-hoc power analysis confirmed approximately 80% power for detecting medium effect sizes, it may still limit the detection of differences in rarer complications. Finally, the lack of follow-up beyond 12 weeks precludes assessment of longer-term sequelae, such as growth disturbances or late deformities.

## Conclusions

This study demonstrates that the walking hip spica cast is a more effective and caregiver-friendly option than the traditional hip spica cast for managing femoral shaft fractures in children under five. It was associated with fewer complications, faster functional recovery, shorter immobilization periods, and greater parental satisfaction. Radiological union was also achieved more promptly, without compromising fracture stability. These findings support the use of the walking hip spica as a preferred conservative treatment approach in this pediatric population.

## Appendices

**Table 7:** Proforma for Data Collection

Variable	Response
Patient ID	
Age (in years)	
Gender	☐ Male ☐ Female
Side of Injury	☐ Right ☐ Left
Fracture Pattern	☐ Transverse ☐ Oblique/Spiral
Mechanism of Injury	☐ Fall from height ☐ Household accident
Type of Spica Cast	☐ Walking Spica ☐ Conventional Spica
Date of Cast Application	
Duration of Immobilization (days)	
Radiological Union (in weeks)	
Cast-Related Complications	☐ None ☐ Skin Excoriation ☐ Pressure Sores ☐ Cast Breakage
Re-displacement Present	☐ Yes ☐ No
Malunion Present	☐ Yes ☐ No
Functional Recovery Milestones	
– Sitting Independently (2 weeks)	☐ Yes ☐ No
– Standing with Support (4 weeks)	☐ Yes ☐ No
– Walking Unassisted (6 weeks)	☐ Yes ☐ No
– Walking Independently (12 weeks)	☐ Yes ☐ No
Parental Satisfaction	☐ High ☐ Low
Difficulty in Hygiene Care	☐ Yes ☐ No
Would Choose the Same Cast Again	☐ Yes ☐ No
Overall Outcome	☐ Excellent ☐ Satisfactory ☐ Poor
